# Domestic wastewater treatment by *Pistia stratiotes* in constructed wetland

**DOI:** 10.1038/s41598-024-57329-y

**Published:** 2024-03-30

**Authors:** Majid Ali, Ambreen Aslam, Abdul Qadeer, Sabiha Javied, Numrah Nisar, Nayyer Hassan, Afzal Hussain, Basharat Ali, Rashid Iqbal, Talha Chaudhary, Mona S. Alwahibi, Mohamed S. Elshikh

**Affiliations:** 1 Environmental Sciences Department, The University of Lahore, Lahore, 54000 Pakistan; 2 Mehran University of Engineering and Technology, Jamshoro, 76060 Pakistan; 3 Lahore College for Women University, Lahore, 54000 Pakistan; 4 English Department, University of Lahore, Lahore, 54000 Pakistan; 5 Department of Agricultural Engineering, Khwaja Fareed University of Engineering and Information Technology, Rahim Yar Khan, 64200 Pakistan; 6 Department of Agronomy, Faculty of Agriculture and Environment, The Islamia University of Bahawalpur, Bahawalpur, 63100 Pakistan; 7 Faculty of Agricultural and Environmental Sciences, Hungarian University of Agriculture and Life Sciences, Godollo, 2100 Hungary; 8 Department of Botany and Microbiology, College of Science, King Saud University, P.O. 2455, 11451 Riyadh, Saudi Arabia

**Keywords:** *Pistia stratiotes*, Constructed wetland, Domestic wastewater treatment, Macrophytes, Removal efficiency, Hydraulic retention time, Plant sciences, Environmental sciences, Hydrology

## Abstract

The objective of the study was to evaluate the performance of *Pistia stratiotes* for treatment of domestic wastewater in a free surface water flow constructed wetland. The objective of the study was to evaluate contaminants removal efficiency of the constructed wetland vegetated with *P. stratiotes* in treatment of domestic wastewater against Hydraulic retention time (HRT) of 10, 20 and 30 days was investigated. This asks for newer and efficient low-cost nature-based water treatment system which along with cost takes into consideration the sustainability of the ecosystem. Five constructed wetland setups improved the wastewater quality and purify it significantly by reducing the TDS by 83%, TSS by 82%, BOD by 82%, COD by 81%, Chloride by 80%, Sulfate by 77%, NH_3_ by 84% and Total Oil and Grease by 74%. There was an increase in pH of about 11.9%. Color and odor of wastewater was also improved significantly and effectively. It was observed that 30 days’ HRT was optimum for the treatment of domestic wastewater. The final effluent was found to be suitable as per national environmental quality standards and recycled for watering plants and crop irrigation but not for drinking purposes. The treatment in constructed wetland system was found to be economical, as the cost of construction only was involved and operational and maintenance cost very minimal. Even this research was conducted on the sole purpose of commuting the efficiency of pollutant removal in short span time.

## Introduction

For many years, a large amount of untreated effluent was dispensed into streams, rivers, lakes, coastal areas and seasonal tributaries. Population growth, anthropogenic activities land, expansion of numerous industries and climatic changes are the main reasons behind the decline and contamination of the aquatic environment^1,2^. When untreated water is directly released into the natural water bodies, it not only destroys the aquatic systems but also spoils the condition of water resources by a large amount. Increased amount of contaminants in the wastewater is extremely dangerous for the existence of aquatic ecosystem and it further increases threats on health of human beings^3–5^. Topical researchers have declared that phytoremediation by aquatic plants have a great potential to eradicate numerous organic and inorganic pollutants from wastewater^6–12^. Application of free-floating aquatic plants for wastewater treatment in the constructed wetlands is a natural, low cost and environment-friendly method for wastewater management^13–16^. Plant roots play a key role in floating treatment wetland (FTW), by providing a living, yet high specific surface area for the development of biofilms that contain diverse micro-organism communities responsible for filtering and entrapping fine suspended particles^17,18^. Constructed Wetlands are artificially engineered treatment systems that utilize natural cycles or processes involving soils, wetland vegetation, and plant and soil-associated microbial assemblages to remediate contaminated water and improve its quality. Herein, we present a detailed assessment of contaminant removal effectiveness in different CW systems, i.e., free-water surface or surface-flow constructed wetlands (FWSCWs/SFCWs), subsurface-flow constructed wetlands (SSFCWs), and hybrid constructed wetlands (HCWs)^19^. Constructed wetlands are designed and built to replica the processes happening in natural wetlands. In this current era, constructed wetlands are broadly used and applied across the world in order to treat wastewater from all sectors for the purpose of wastewater management^20,21^.

Pakistan is an agricultural country and most of the economy depends on water for growing crops. It is the third most affected country in terms of water scarcity as per International Monetary Fund^22^. Presently, only 20% population of the country has access to clean drinking water. The rest 80% population has no availability to clean water and has to depend on polluted and contaminated water from different sources such as sewage, industrial effluents, pesticides and fertilizers etc.^23,24^. Therefore, in view of wastewater management and water sustainability, it is the only option left that water is recovered and recycled to meet the future water shortage challenges. The effluent from the wet land was found useful in irrigation and keeping aquatic animals. However, it was noted that a 30- day detention time was found optimized for the treatment kitchen waste water^25^.

In major cities, domestic and industrial effluents are discharged into natural tributaries, streams, lakes, canals, and rivers, being the instantaneous discharging vicinity close to the cities resulting high pollution load in the water bodies. Pakistan is a developing country and cannot afford expensive conventional technologies to treat wastewater. It has a suitable climate and land availability for wastewater treatment by applying constructed wetlands in the peripheries of cities and towns, as it can provide a cost-effective, long lasting and aesthetic solution to wastewater remediation. CW have the potential to offset energy and irrigation needs at scales ranging from small communities to peri-urban areas. Constructed wetlands used to treat wastewater have the potential to provide a sustainable bioenergy source without placing burdens on water resources or displacing other food or energy crops^26^.

*Pistia stratiotes* is a kind of free-floating aquatic macrophytes which is broadly applied for the elimination of containments from the wastewater prior to discharge in order to decrease the pollution loads on natural water bodies. The biomass of the *P. stratiotes* increases rapidly. It is reported that in just 5, 10 and 20 days its mass becomes double, triple, and quadruple respectively. In a period of less than 1 month its biomass will increase by nine times as compared to its original biomass. It possesses long hanging root-biofilm network as biologically active surface area for biochemical and physical processes such as filtering and entrapment. These submerged long roots capitalize on the contact between the root-biofilm network and the contaminated and polluted water passing through the constructed wetlands. The reason for choosing *P. stratiotes* is its high pollutant removal efficiency and it can also remove organic and inorganic pollutants including heavy metals from the industrial, domestic, municipal, agricultural sewage sludge, and drainage ditches^16,27^. Despite its notorious reputation as an alien plants, water lettuce has also been widely applied in wastewater phytoremediation in tropical areas^28^ because of its prolific growth characteristics^29^, great potential in nitrogen and phosphorous removal^30^, significant absorption, and enrichment in several heavy metals^31^.

A larger quantity of *P. stratiotes* plants translates to a higher total surface area of roots and leaves available for nutrient and pollutant absorption. The increased surface area provides more opportunities for the plant to interact with and uptake contaminants from the water. Further, raw water first stored in septic tanks from where water is pumped into constructed wetlands (CWs) by pumping unit. So, treatment of raw water also took place in septic tanks to some extents. The objective of the present study was to evaluate the removal efficiency of a constructed wetland using *P. stratiotes* in the treatment of domestic wastewater. Therefore, a free water surface flow constructed wetland was built by a multinational oil and gas organization in its facility near Gumbt, district Kohat, Khyber Pakhtukhwa, Pakistan to treat domestic wastewater. The research is significant for improving the overall efficiency of constructed wetlands by harnessing the pollutant removal capabilities of *P. stratiotes*. This can contribute to more sustainable and environmentally friendly wastewater treatment practices. Various physico chemical parameters such as biochemical oxygen demand (BOD_5_), chemical oxygen demand (COD), total dissolved solids (TDS), total suspended solids (TSS), sulfate, chloride, ammonia, color and pH were analyzed in the laboratory in order to check the quality of water after phytoremediation. The research contributes to the scientific understanding of the interactions between *P. stratiotes* and pollutants in constructed wetlands. It adds valuable knowledge to the field of phytoremediation and sustainable water management practices.

## Results and discussion

**Figure 1:**
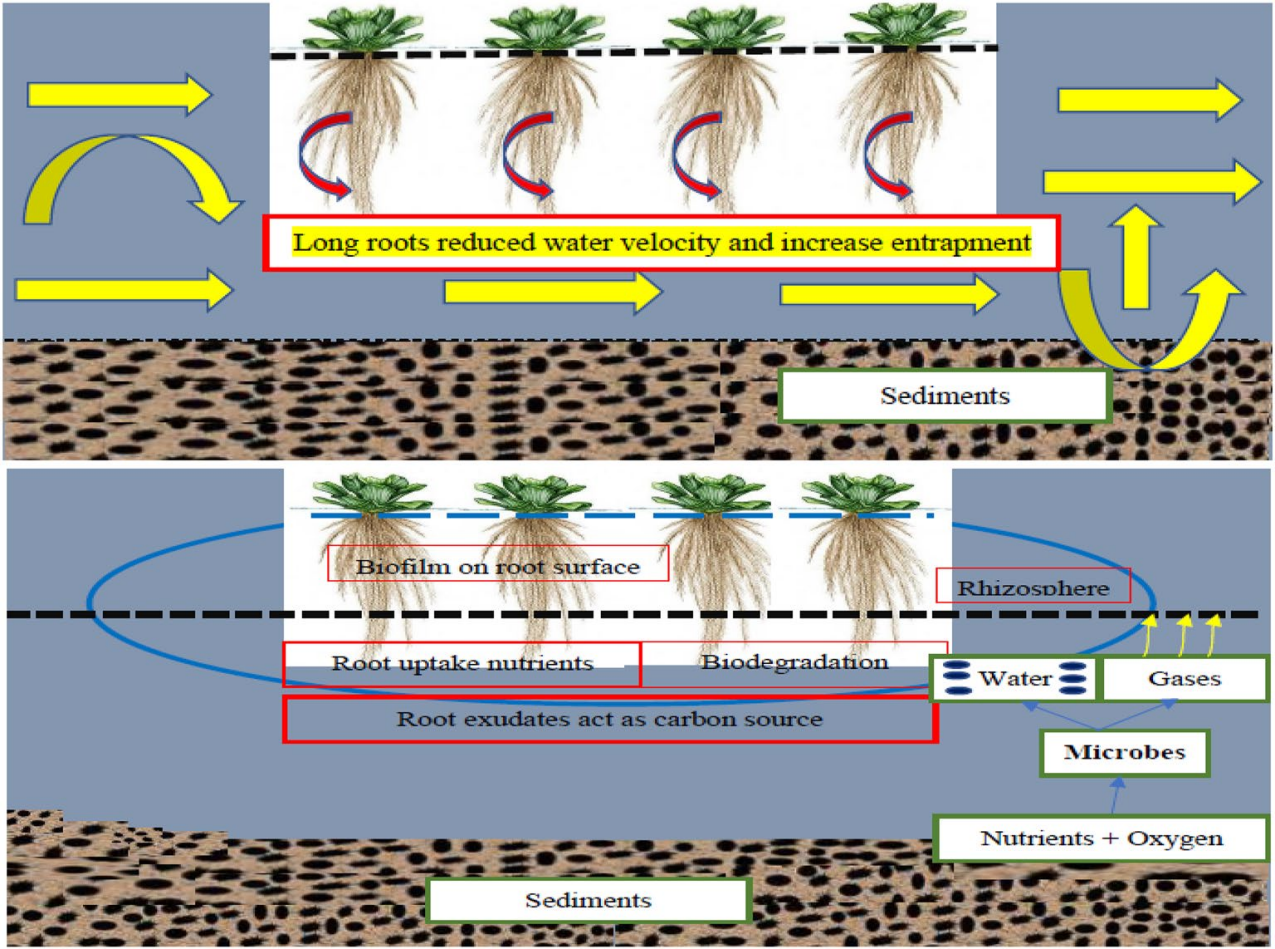
*Pistia stratiotes* hanging root system and purification mechanism in constructed wetland.

Sedimentation, filtration, deposition, adsorption, and microbial mediated reactions and *Pistia stratiotes* uptake, transport, and biological assimilation are the processes involved in the removal of pollutants. *Pistia stratiotes* has a hanging network of long roots, rhizomes and attached biofilm on the root surfaces. This hanging root-biofilm network of *Pistia stratiotes* provides a biologically active surface area for biochemical and physical processes such as filtering and entrapment. These submerged long roots capitalize on the contact between the root-biofilm network and the contaminated and polluted water^32,33^. These roots also reduce the water velocity which ultimately enhances the microbial activities, sedimentation, plant uptake, transport and biological assimilation rates, thus enhancing pollutant removal efficiency (Fig. 1).

**Figure 2:**
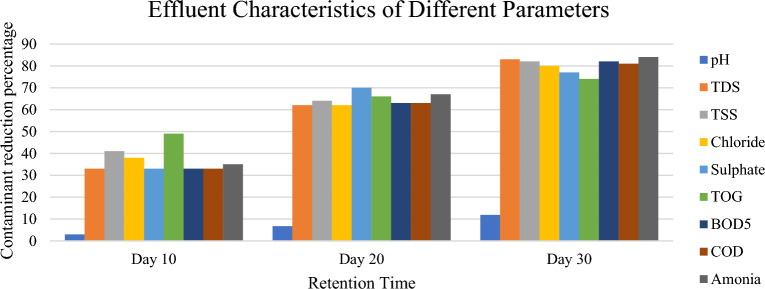
Containments % reduction against HRT.

In Fig. 1, the plant floats on the water surface, and its roots dangle beneath, submerged in the water column. These roots are feathery and have a high surface area, facilitating efficient nutrient and pollutant absorption. The characteristics of this root system and its interactions with pollutants is essential for optimizing the performance of constructed wetlands for wastewater treatment. Figure 2 (Table 1) is showing the reduced percentage of containments against HRT.

**Table 1:** Containments (%) reduction against HRT.

Parameters	Day 10	Day 20	Day 30
pH	2.9	6.7	11.9
TDS	33	62	83
TSS	41	64	82
Chloride	38	62	80
Sulphate	33	70	77
Total oil and grease	49	66	74
BOD5	33	63	82
COD	33	63	81
Ammonia	35	67	84

### Biological oxygen demand and chemical oxygen demand

**Figure 3:**
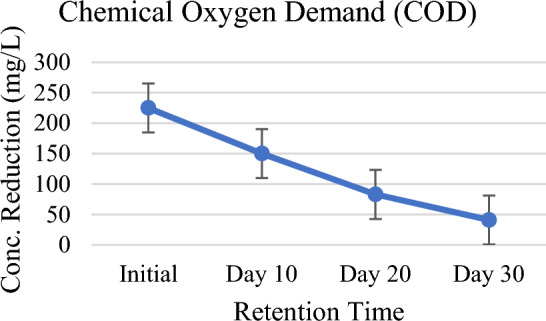
Chemical oxygen demand (COD) against HRT.

**Figure 4:**
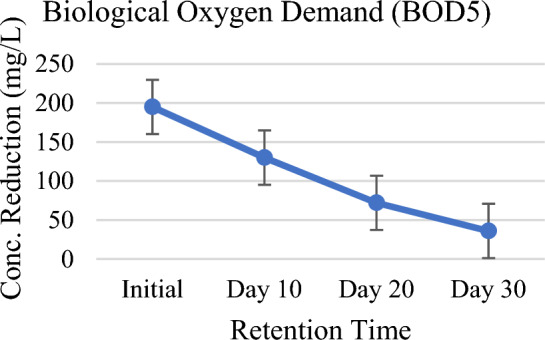
Biological oxygen demand (BOD5) against HRT.

The result of the analysis has shown progressive and consistent reductions of BOD and COD levels in wastewater. There was 82% of BOD and 81% of COD reduction on 30 days which is the maximum as per the record (Figs. 3, 4). Rise in pH (Fig. 11) has also confirmed their removal in the constructed wetland vegetated with *P. stratiotes.* Chandanshive et al.^34^ have already proved in a research study that pH of the wastewater can be increased by applying free floating aquatic macrophytes by reducing BOD and COD in wastewater. The removal of chemical oxygen demand by *A. filiculoides* (96%) was slightly higher than the *L. minor* (92%), but the biochemical oxygen demand removal by *L. minor* (92%) was significantly higher than *A. filiculoides* (90%). Despite the high removals of chemical and biochemical oxygen demands, total phosphorus and total nitrogen attained, the concentrations were found exceeding the discharge and agricultural reuse limits^35^. Similar results have also been investigated and reported that higher COD and BOD levels can be degraded by using different free-floating aquatic macrophytes to treat different wastewaters^36,37^. Free floating aquatic plants support the development of microorganisms and their activities which provides the foundations for decomposing organic matters^38–40^. The only demerit of *P. stratiotes* is that it does not grow at higher COD levels^41^.

### Total suspended solids and total dissolved solids

**Figure 5:**
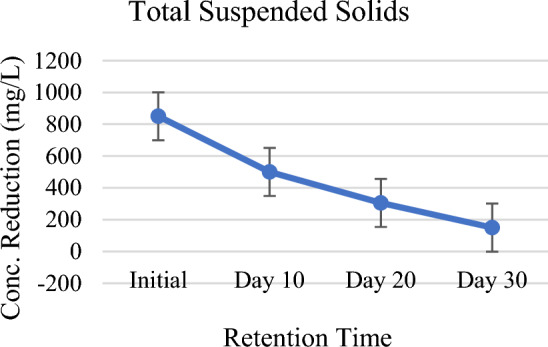
Total suspended solid against HRT.

**Figure 6:**
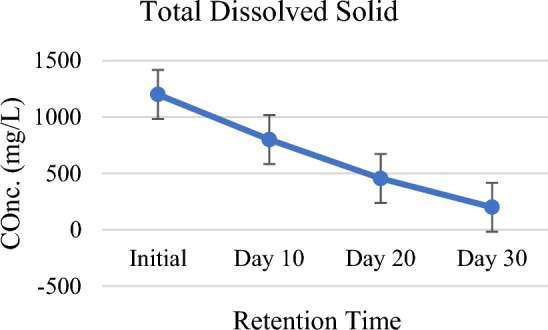
Total dissolved solid against HRT.

Reduction trend in the levels of TSS and TDS has been continuously observed in the setup against the HRT. The results showed TSS and TDS levels as 850 mg/L and 1200 mg/L for raw water but for the HRT levels are 500 mg/L (reduced by 41%), 305 mg/L (reduced by 64%), 150 mg/L (reduced by 82%), 800 mg/L (reduced by 33%), 455 mg/L (reduced by 62%) and 200 mg/L (reduced by 83%) respectively (Figs. 5, 6). Insistent reduction was because of the sedimentation, filtration, deposition, adsorption, and microbial activities in the constructed wetland. The rate of microbial activities was highest in the pond 5 where large number of *P. stratiotes* were planted. Da Silva et al.^42^ and Osti et al.^43^ have already proved in a study that TSS and TDS reduction by *P. stratiotes* is higher than all other free-floating aquatic macrophytes. *Pistia stratiotes* and *Chrysopogon zizanioides* for the removal of Total dissolved solids (TDS) from second stage RO brine solution of textile dying industry by attached growth shallow pond system. Under 10 days of operational period, water hyacinth (W.H) showed maximum removal of 55.6% of TDS on 6th day, 48.7% in case of water lettuce (W.L) on 7th day and 39.6% TDS removal on 3rd under Vetiver (V.V) treatment system^44^. Treatment system also reduced total dissolved solids (TDS) around 35.2% by combined effect of wetland plants namely *L. minor, T. latifolia*, and *S. acutuson*. Sequential phytoremediation with a mixture of plants was more effective than that relying only on a single plant species^45^. In addition Lu et al.^30^ observed that total suspended particles in the water column were reduced by around 10% in treatment plots as compared to control plots.

### Ammonia

**Figure 7:**
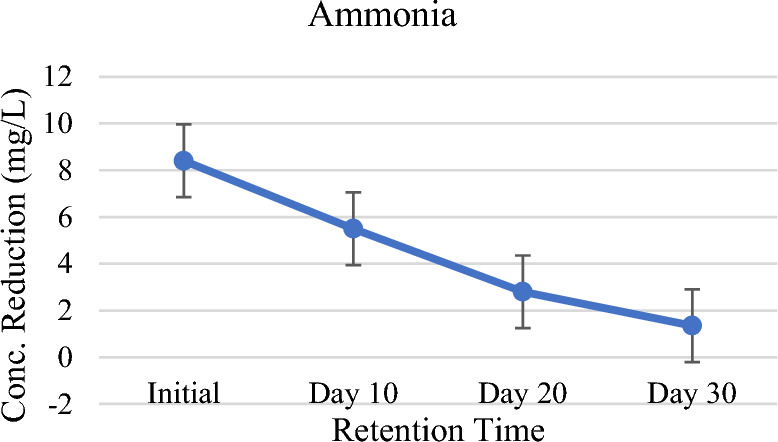
Graph of ammonia against HRT.

Both ammonia (NH_3_) as well as ionized ammonia (NH_4_^+^) exist in water in a soluble form in a state of equilibrium. Ammonium is a vital nutrient needed for free-floating aquatic macrophytes growth like *Pistia stratiotes*. Fresh sewage has around 25% nitrogen in organic form. Environmental impacts of ammoniacal nitrogen in wastewater are acidifications, aquatic ecosystems variations, and eutrophication of surface water^46^. Therefore, it is very necessary to treat the excess ammonia in the wastewater prior to discharge into the aquatic environment. The nitrogen in the form of organic is almost converted to NH_3_-N, and further converted to nitrate with the aid of microbial oxidation. *Pistia stratiotes* is reported to reduce the ammonium ions from the water as it utilizes NH_4_–N prior to NO_3_–N as nitrogen source and does not switch on the utilization of NO_3_–N until NH_4_–N gets consumed entirely^47^. Shah et al.^48^ has also reported a similar investigation in an experimental study. The results confirmed that *P. stratiotes* remarkably reduced ammonia from initial levels of 8.4 mg/L to final 1.35 mg/L (Table 2, Fig. 7). *P. stratiotes* absorb the nutrients through roots and transport them to other parts of the plant for various cellular functions. The uptake, transportation, and assimilation in different body parts are the steps involved for ammoniacal nitrogen utilization in macrophytes^49,50^. Aquatic macrophytes use ammoniacal nitrogen for plant growth^51^. In addition to plant uptake, removal of N also occurred by volatilization of NH_3_ with increasing pH in the water. Ammonia removal can also occur by nitrification and denitrification under aerobic and anaerobic conditions. Shah et al.^48^ investigated in a study that higher values of pH in wastewater favor the volatilization of NH_3_. Kutty et al.^52^ reported the ability of water hyacinth for removing ammonia, phosphorus and nitrate from the municipal wastewater treatment plant effluent. Constructed wetlands are almost completely conversed with emerging macrophytes and are being managed as water quality improving systems, some commonly used macrophytes.

**Table 2:** Containments reduction against HRT.

Parameters	Initial	Day 10	Day 20	Day 30	Units
pH	6.9	6.9	7.15	7.5	–
TDS	1200	800	455	200	mg/L
TSS	850	500	305	150	mg/L
Chloride	200	155	95	50	mg/L
Sulphate	1000	667	300	227	mg/L
Total oil and grease	98	50	33	25	mg/L
BOD5	195	130	72	36	mg/L
COD	225	150	83	41	mg/L
Ammonia	8.4	5.5	2.8	1.35	mg/L

### Sulfate and chloride

**Figure 8:**
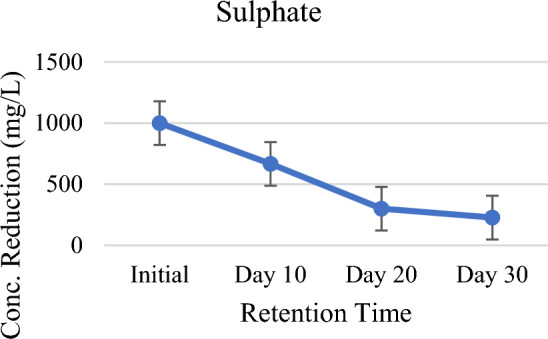
Graph of sulfate against HRT.

**Figure 9:**
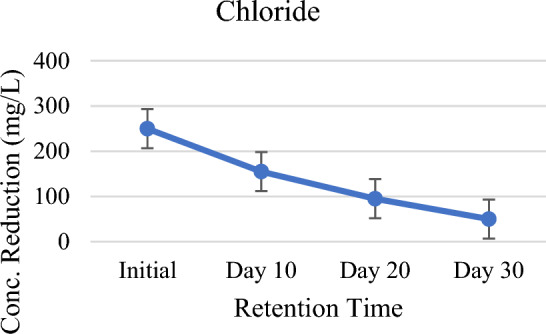
Chloride against HRT.

As shown in Table 2 and Fig. 8, at the end of retention period that the system effectively and consistently removed 33%, 70% and 77% of Sulfate (SO_4_^2−^). In the setup, vegetated *P. stratiotes* provided an appropriate roots zone for biological assimilation and the sulfate precipitation process. Redox state dynamics at the roots zone of *P. stratiotes* together with organic carbon and O_2_ is responsible for the fate of sulfate in the wastewater^53^. Microbes especially sulfate-reducing bacteria, play vital role in the reduction of sulfate in the presence of oxygen^54^. Moreover, SO_4_^2−^ loses due to oxidation of sulfide (S^2−^) to SO_4_^2−^, and by dissociation reactions in which S^2−^ is converted into hydrogen sulfide. Hydrogen sulfide is chiefly volatile to atmosphere^55^. At the initial stage, rise of pH indicate that hydrogen sulfide has emitted to the atmosphere. Formation of insoluble metal sulfates (like FeSO_4_, ZnSO_4_) and mineral sulfates (such as CaSO_4_) caused by heavy metal precipitation are other sources of sulfate losses. This happens when SO_4_^−2^ is more auspicious than S^−2^ in wastewater^56^. Macrophytes and microbe’s uptake organic S for their biomass^3^, O_2_ released by the plants via roots zone utilized for direct re-oxidation of S^−2^ to SO_4_^−2^, stimulate microorganism growth and activity^57^. Thus, it can be concluded that uptake by *P. stratiotes*, sulfate precipitation, reduction–oxidation of S^−2^ in the system were the processes involved in the removal of SO_4_^−2^. In case of chloride reduction in chloride observed was 80% at the 30-day of HRT (Fig. 9). Chloride is required in minor amounts and it helps in processes such as growth, photosynthesis, metabolism, photosynthesis, osmosis, and ionic balance within the cells of *Pistia stratiotes*. High reduction of chloride might happened be due to its minute concentration in wastewater^51^.

### Total oil and grease

**Figure 10:**
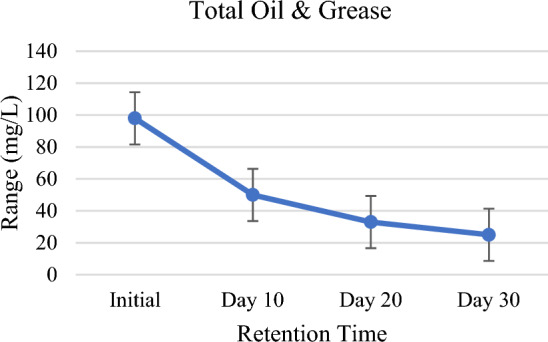
Total oil and grease against HRT.

The result obtained shows a consistent pattern for the removal of total oil and grease from the wastewater in the constructed wetland planted with *P. stratiotes*. The initial value of oil and grease in wastewater is 98 mg/L which has reduced to 50 mg/L (49% reduction), 33 mg/L (66% reduction), and 25 mg/L (74% reduction) in the system for the HRT of 10, 20 and 30 days (Table 2, Figs. 2, 10). It has already been reported that degradation of oil and grease by macrophytes is attributed to the enhancement of microbial growth resulting in reduction of oil and grease^58,59^.

### Odor

Odor of domestic wastewater was offensive due to variation of organic and inorganic pollutants, which lower atmospheric ambient air quality and is a health hazard for nearby community. *Pistia stratiotes* in the system, progressively and effectively reduced the offensive odor to odorless in the HRT of 10, 20 and 30 days by incessant effective eradication of organic and inorganic pollutants in the wastewater. Oladejo et al.^60^ has already confirmed that *Pistia stratiotes* has the capability to change offensive odor of wastewater to odorless.

### Color

One of the most important parameters present in domestic wastewater is color. It is caused by variations in contaminants and their mutual reactions in wastewater. *P. stratiotes* has changed the color of wastewater from grey black to colorless. The significant improvement in the color of wastewater indicated a significant elimination of various pollutants from the wastewater by *P. stratiotes.* The color elimination capability of *P. stratiotes* might be attributed to the appropriate particle sedimentation capability of *P. stratiotes* or the capacity of the root of *P. stratiotes* to hold together coarse and fine particles^61^.

### pH

**Figure 11:**
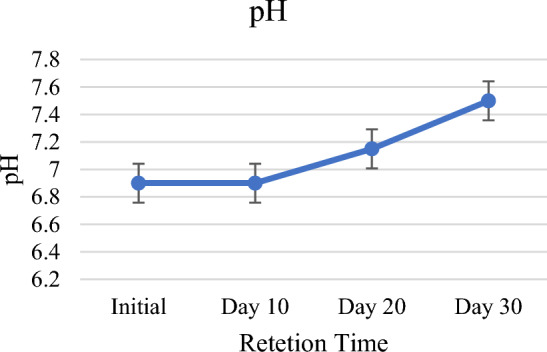
Graph of pH against HRT.

The pH of raw water was slightly acidic, the results obtained have shown that *P. stratiotes* in the setup has improved the pH of the wastewater from 6.7 to 7.5 (Table 2, Fig. 11). The highest increased pH 11.9% was observed on 30-day (Table 2, Fig. 2). The pH of the domestic wastewater was improved from slightly acidic to slightly basic. This rise in pH might occurred be due to the consumption of carbon dioxide by the plants during the process of photosynthesis. Irawati et al.^62^ has already reported that an increase in pH might be accredited to the consumption of CO_2_ during the process of photosynthesis by macrophytes. Similar results regarding the rise in pH have been reported by Galal et al.^63^, Polechońska et al.^64^ and Zhao et al.^65^ in their research studies carried out on free floating aquatic macrophytes for different wastewater streams. Also, the growth of water lettuce resulted to a fall in the pH of the water, even though it was not expected because usually, plant photosynthesis raises the pH of water^66^. According to Raju et al.^67^, the maximum increase in biomass production occurred at neutral and slightly alkaline pH (pH 7 and 8), which may be due to the increase in nutrient uptake and immobilization capacity of the water hyacinth samples. The pH of influent water ranged from pH 7.0 to 8.7, and the suitable pH controlled the nutrient absorption and biochemical reactions that took place in living organisms^68^.

## Materials and methods

### Study area

A free water surface flow constructed wetland was built by an oil and gas exploration and production multinational organization at its facility to treat domestic wastewater as per National Environmental Quality Standards (NEQS) near town Seni Gumbat, located in Kohat District of Khyber Pakhtunkhwa, Pakistan which lies between latitude 33.5110544° N and longitude 71.709795° E. The terrain consists of mountain ranges, undulating sub montane areas, and plains surrounded by hills. In the north, the mountain ranges generally run north–south; south of the Kābul River, which bisects the province from east to west, the ranges generally run east–west. Kohat valley has a hot semi-arid climate. The district’s yearly temperature and precipitation is 19.15 °C and 128.57 mm respectively.

### Constructed wetland setup

Mechanical excavation was carried out to dig a basin of 2.7 m deep. The first layer at the basin is of 2.43 m stone soling followed by 17 cm plain cement concrete (PCC) and high-density polyethylene liner was spread over PCC layer to avoid leaching and infiltration of wastewater down the soil in order to avoid contamination of underground water in case of seepage and leakage. The basin has been divided into six sections by 2.1 m high and 2.7 m wide brick walls namely sedimentation pond with dimension 9.4 m × 6.7 m × 2.1 m and five ponds each with dimension 7 m × 6.7 m × 2.1 m interconnected with three holes each of 2.43 m size in the brick walls to provide maximum HRT for wastewater in each section.

**Figure 12:**
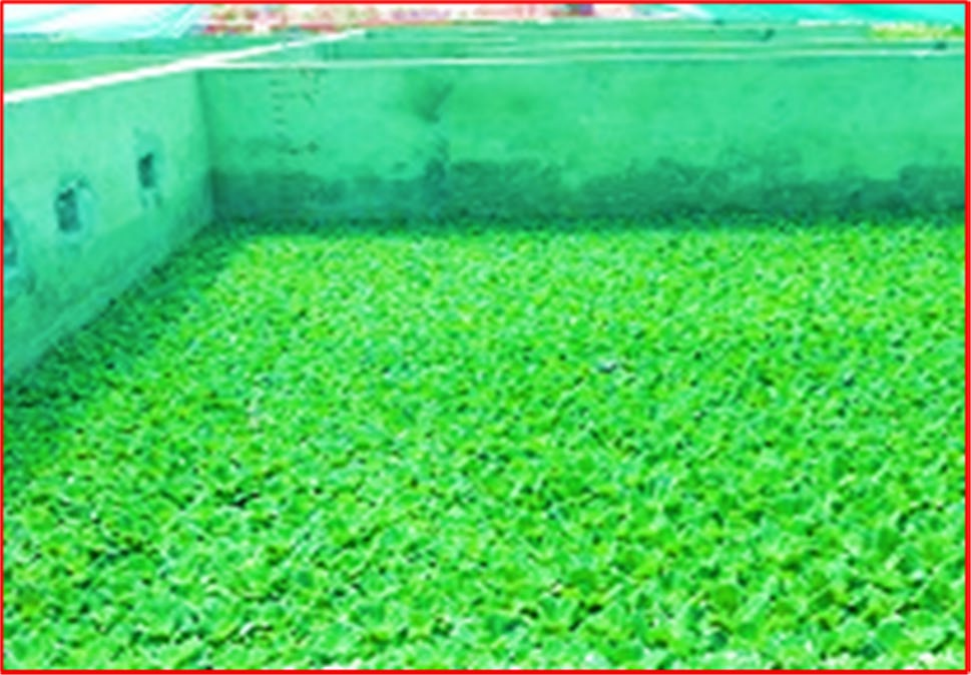
*P. stratiotes* growth in the wetlands.

**Figure 13:**
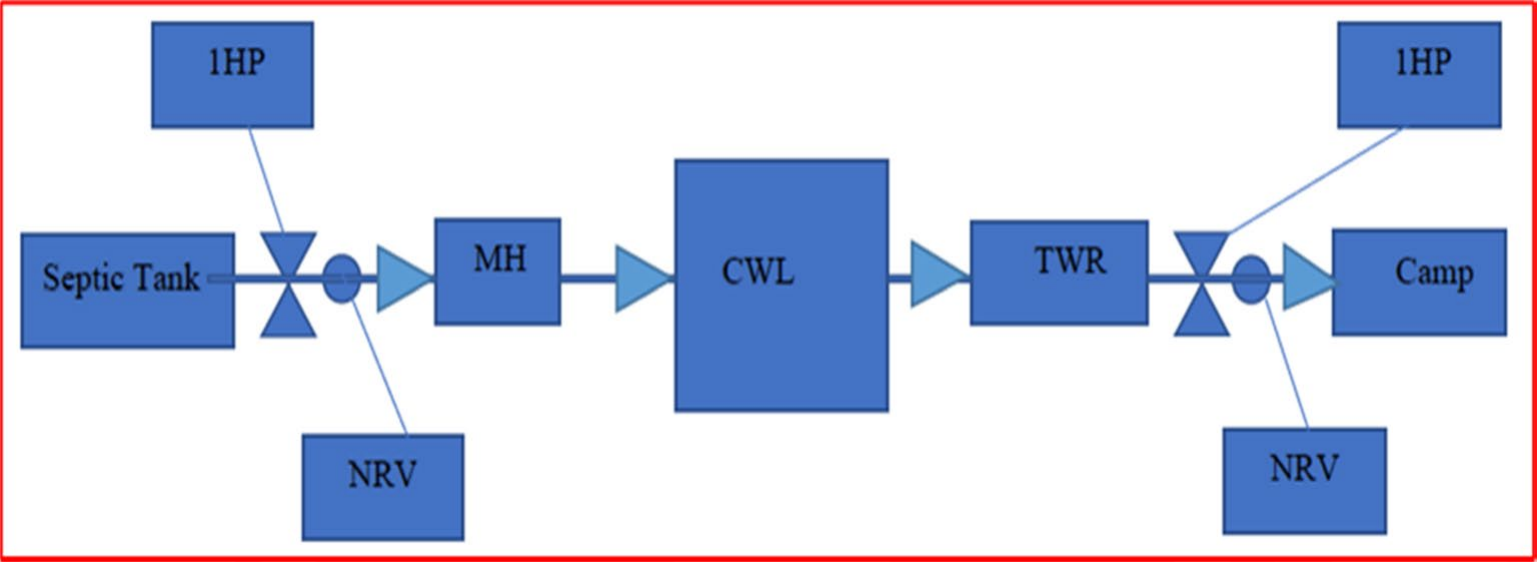
Schematic flow of wastewater in the constructed wetlands.

**Figure 14:**
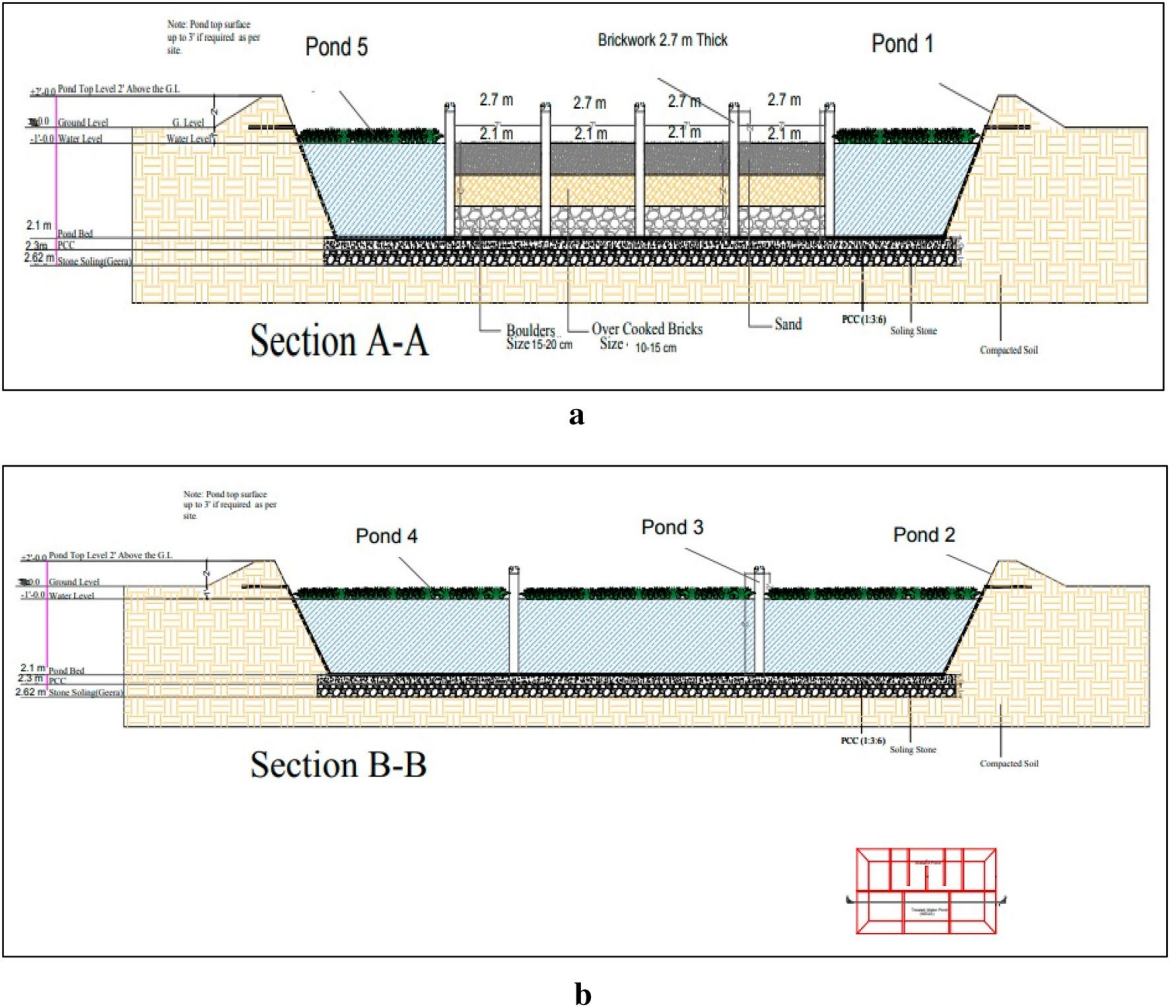
(**a**) Design of constructed wetland experimental setup. (**b**) Drawing shows the dimensions of all wetlands.

Screening unit is applied at each primary wastewater generation entity like kitchens, messes, showers, wash basins, toilets, laundries, and vehicle wash station to avoid wastewater free from any dirt, debris and littering of all kinds as low as reasonably practicable especially non-biodegradable materials. The wastewater is collected in the septic tank for settling of sludge, heavy particles, decomposition, and reduction of organic matter by anaerobic bacteria and is pumped by a suction pump of I horsepower (HP) fitted with NRV (no return valve) into the manhole close to the constructed wetland at the rate of 2.5 m^3^/h. The wastewater from the manhole flows in the sedimentation pond provided with filter bed consists of a layer of 0.6 m boulders, 0.6 m overcooked bricks and 0.6 m sand throughout its bed. The size of boulders is 15 cm to 20 cm, 10 cm to 15 cm of overcooked bricks. Overcooked bricks can be utilized as a feasible alternative to natural pebbles. Filter bed with dimension 8.8 m × 6.7 m × 2.1 m increases efficiency for trapping the suspended particles in the wastewater and also acts as substrate for physical, chemical, biological processes and microbial activities to remove pollutants. Turbidity removal efficiency increased with the increment of influent turbidity when bricks were used as the filter media. Valves are control points to control water flow on need basis as a safety back up. Results were not calculated from basin to basin but finally from treated water reservoir (last basin separately constructed not part of CWs. This section is further divided into four parts separated by 2.1 m high and 2.7 m wide brick walls, each wall has three 20 cm holes for flow of wastewater from one section to another so as to enhance the HRT within the sections. The purpose of inserting walls is to increase HRT. Numerous *P. stratiotes* are planted in these sections to upsurge microbial activities against the suspended particles settled in sections and trapped in filter bed. Water from the sedimentation pond flows in pond 1, 2, 3, 4 and 5 being interconnected and planted with a large number of *P. stratiotes*. In these ponds, Phytoremediation process would be at utmost stage with free floating aquatic plants*.* Water treated from these ponds finally flows into the treated water reservoir, a separate constructed tank connected with pond 5 by Poly Vinyl Chloride pipe. Treated water at this stage usually displays quality parameters according to NEQS. It flows by gravity or it can be pumped with 1HP suction pump fitted with NRV (no return valve) into the distribution network (thirty-eight distribution units fitted with valves) installed in the facility at different locations for watering plants, orchards, grass ground of the facility. The schematic flow of wastewater and various sections of setup are shown in (Figs. 12, 13, 14).

### Selection of species of macrophytes

*P. stratiotes* were selected for the study keeping in view the accessibility of plants as well as the environmental conditions of the area. Plants were provided by Nano Bio-Solution (Pvt), limited Islamabad (Wastewater treatment services provider). At least hundred plants were inserted in each pond and in filtration bed as well. Plant size was 4inch which increased in size in days due to absorption of nutrients from domestic waste water.

### Sample collection and analytical procedures

50 mL of small samples were collected at regular intervals, combine them, and mix thoroughly to create a composite sample. Samples were collected only one time after the retention time (HRT) of 10 days, 20 days, and 30 days. The constructed wetland took one month for filling after retaining water for long run. The physico-chemical parameters were measured in situ by using a multi-probe digital meter, BOD/COD meters and atomic absorption. The quantitative analysis of the collected samples was carried out according to APHA (2017) to determine the effect of HRT on the wastewater quality. The parameters tested for are: Colour, odour, pH, Temperature, TDS, TSS, Biochemical Oxygen Demand (BOD5), Chemical oxygen demand (COD), Chloride (Cl^−^) and Sulfate (SO_4_), total O&G (TOG) and Ammonia.

Characteristics of raw wastewater and treated water are summarized in Table 3.

**Table 3:** Physicochemical analysis of wastewater during HRT.

Parameters	Initial levels	HRT (days)			Units
		10	20	30	
Color	Grey Black	–	–	Colorless	NA
Odor	Offensive	–	–	Odorless	NA
pH*	6.7	6.9	7.15	7.5	–
TDS	1200	800	455	200	mg/L
TSS	850	500	305	150	mg/L
Chloride	200	155	95	50	mg/L
Sulfate	1000	667	300	227	mg/L
Total O&G	98	50	33	25	mg/L
BOD	195	130	72	36	mg/L
COD	225	150	83	41	mg/L
Ammonia	8.4	5.5	2.8	1.35	mg/L

*Value increased.

Statistical analysis was used to process the data, such as mean, standard error, removal efficiencies, linear correlation, and analysis of variance (One-way ANOVA) was done, to analyze efficiency of constructed wetland to treat the wastewater (Table 4). The P-value was 0.000454 and F crit was 2.305 within the group when considered it as source of variance.

**Table 4:** Statistical analysis of the data (sum, average, variance).

Groups	Count	Sum	Average	Variance
pH	4	28.45	7.1125	0.080625
TDS	4	2655	663.75	188,256.3
TSS	4	1805	451.25	91,172.92
Chloride	4	550	137.5	7475
Sulphate	4	2194	548.5	127,669.7
Total oil and grease (TOG)	4	206	51.5	1069.667
BOD_5_	4	433	108.25	4844.25
COD	4	499	124.75	6481.583
Ammonia	4	18.05	4.5125	9.673958

### Statement on guidelines

All experimental studies and experimental materials involved in this research are in full compliance with relevant institutional, national and international guidelines and legislation.

## Conclusion

The results of the study have revealed that removal efficiency of *P. stratiotes* based constructed wetland was found to be 83% for TDS, 82% for TSS, 82% for BOD, 81% for COD, 80% for Chloride, 77% for sulfate, 84% for NH_3_ and 74% for total oil and grease respectively. Sedimentation, filtration, deposition, adsorption, and microbial mediated reactions, formation of solid compounds, and *P. stratiotes* uptake were contaminants removal mechanisms in the system. The constructed wetland vegetated with *P. stratiotes,* if appropriately managed, are helpful in dropping the level of organic and inorganic constituents of waste water, before being discharged into drainage system to prevent effects on aquatic flora and fauna. It also minimizes the risk of underground water contamination. Furthermore, it is need of hour that constructed wetland system vegetated by macrophytes should be used for treatment of wastewater being eco-friendly and to have a low O&M cost. The use of *P. stratiotes* and the edible plant *L. sativa* could be a potential option to treat domestic wastewater due to relatively high nutrient and organic matter removal efficiency. It is recommended to acclimatize plant for some time after being moved from its original location. For maintenance purposes, the leaves of the plant should be collected regularly to ensure active plants shoot system which enhance proper metabolic activities and nutrient absorption.

## Data Availability

The datasets analysed during this study are included in this manuscript.
